# Supplementary material for: SARS-CoV-2 and Companion Animals: Sources of Information and Communication Campaign during the COVID-19 Pandemic in Italy
Source: Vet Sci. 2023 Jun 30;10(7):426. doi: 10.3390/vetsci10070426 (PMC10386290; doi:10.3390/vetsci10070426)
Supplement: Supplementary file 1 [file vetsci-10-00426-s001.zip › Table S1.docx]

**QUESTIONNAIRE**

**COMIS - Improving Communication for Public Health in the post-COVID-19 period**

## RELATIONSHIP BETWEEN HUMANS AND COMPANION ANIMALS

**Do you own one or more companion animals?**

| Yes | [1] |
| --- | --- |
| No | [0] |

**Do you work in strict contact with companion animals?**

| Yes | [1] |
| --- | --- |
| No | [0] |

**Do you know that companion animals can be infected by Sars-CoV-2?**

| Yes | [1] |
| --- | --- |
| No | [0] |

**Do you know the preventive measures to adopt when a COVID-19 positive person is in contact with a companion animal?**

| Yes | [1] |
| --- | --- |
| No | [0] |

**Do you know which companion animals are more at risk of infection by Sars-CoV-2?**

| Yes | [1] |
| --- | --- |
| No | [0] |

**Where did you find information on COVID-19 and companion animals?** *(only one answer - turn the list)*

| Through television and/or radio broadcasts | [1] |
| --- | --- |
| Through newspapers (printed or online) | [2] |
| On institutional websites (Italian Ministry of Health, etc.) | [3] |
| On social media of friends and/or acquaintances | [4] |
| Through physicians | [5] |
| Through veterinarians | [6] |
| Through friends and relatives | [7] |
| Other | [8] |
| I did not inform myself | [0] |

**Concerning the preventive measures to adopt with companion animals, which of these sources do you trust the most?** *(only one answer - turn the list)*

| Indications from institutions (Italian Ministry of Health, etc.) | [1] |
| --- | --- |
| My physician | [2] |
| My veterinarian | [3] |
| Newspapers | [4] |
| Television and/or radio broadcasts | [5] |
| Friends and/or relatives | [6] |
| Pharmacists | [7] |
| Web forum and/or social media | [8] |

**How much do you agree with each of the following statements about COVID-19 and companion animals?** *(only one answer per row; turn the items order; I don’t know only on interviewed person’s request)*

|  | **A lot** | **Enough** | **Little** | **Not at all** | *I don’t know* |
| --- | --- | --- | --- | --- | --- |
| The transmission of the virus can occur from companion animals to humans | [4] | [3] | [2] | [1] | [99] |
| Humans can transmit the virus to companion animals | [4] | [3] | [2] | [1] | [99] |
| Several dogs and cats become ill after close contact with ill people | [4] | [3] | [2] | [1] | [99] |
| The communication on COVID-19 and companion animals has been inadequate | [4] | [3] | [2] | [1] | [99] |

# DEMOGRAPHIC DATA

**Gender**

| Male | [1] |
| --- | --- |
| Female | [2] |

**Age (year): ________**

**What is your level of education?**

| No education or primary school | [1] |
| --- | --- |
| Secondary school | [2] |
| High school 2-3 years (professional qualification) | [3] |
| High school 4-5 years (diploma) | [4] |
| Undergraduate or post-graduate university degree | [5] |
| *No answer – only on interviewed person’s request* | [99] |

Geographical area

| Northwest | [1] |
| --- | --- |
| Northeast | [2] |
| Centre | [3] |
| South | [4] |
| Islands | [5] |
